# Letter to the Editor

**DOI:** 10.31662/jmaj.2018-0018

**Published:** 2018-09-28

**Authors:** Toshihiko Hasegawa, Toru Kakuta

**Affiliations:** 1 Future Health Research Institute; 2 Public Health Committee of Japan Medical Association

**Keywords:** New health concept, Healthy life expectancy, Super-aged society, WHO definition of Health, World Medical Association

This letter is to reveal the recent development in the new health concept and health indices.

Japan is now leading the world in aging ^(1)^. The percentage of population aged over 65 years is 25.9%, which is by far the world’s highest in 2015 and is 3.8% higher than 2^nd^ placed Italy. According to the Japanese government and UN estimate of 2017, the age structure of the world’s population will be completely different after 45 years ^(1)^ (Figure 1). The super-aged society, defined by WHO as the percentage of population aged over 65 years, is over 21 in only 3 of 201 countries in 2015 but will increase to 100 countries, half of the world, in 2060.

**Figure 1.**
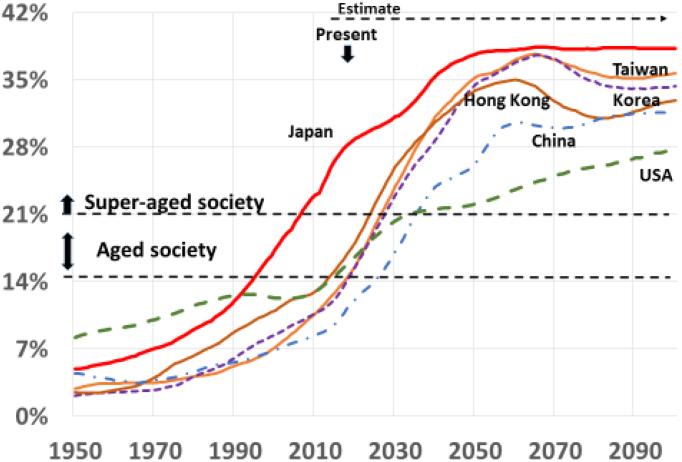
Rate of population aged over 65 in East Asian countries & USA Japanese Government Estimate & UN Estimate, 2017.

The health concept should be changed for the elderly because it is difficult to cure diseases and prevent death during the late stage of life. New health measurements are needed for health management.

With this historical context, Dr. Yokokura, President of the Japan Medical Association, consulted with the Public Health Committee on the new health concept and its measurement in December 2016. The committee reported the result in May 2018 ^(2)^.

The report consisted of background analysis, the new definition of health, challenging the old definition by WHO in 1946, the measurement method of healthy life expectancy, and their applications to healthcare.

The concept of health was examined through multiple perspectives, including etymology, medical anthropology, sociology, philosophy, and history. The modern concept of health was developed in Europe and translated into Japanese in approximately 1830, which is less than 200 years ago ^(3)^. The most frequently cited definition of WHO has been criticized because it was unpractical and unsuitable to the elderly ^(4)^. A historical study indicated that this definition was proposed by the League of Nations officials in 1946 as the part of the “Positive Health Movement” and was the political propaganda for the new world order with young populations after WWII ^(5)^.

The report proposes multidimensional definitions because goal and meaning of heath is different, depending upon the generation or profession. For healthy life expectancies, the report proposes to use the ADL data from the assessment for eligibility of long-term care insurance because it is available at the municipality level from the year 2000.

Dr. Yokokura is expected to lead the historical innovation of new health care for the 21^st^ century based on the new concept not only as the President of the Japan Medical Association but also as the President of the World Medical Association.

## Article Information

### Conflicts of Interest

None
